# Accuracy of computer-aided static and dynamic navigation systems in the placement of zygomatic dental implants

**DOI:** 10.1186/s12903-023-02856-9

**Published:** 2023-03-15

**Authors:** Juan Ramón González Rueda, Agustín Galparsoro Catalán, Víctor Manuel de Paz Hermoso, Elena Riad Deglow, Álvaro Zubizarreta-Macho, Jesús Pato Mourelo, Javier Montero Martín, Sofía Hernández Montero

**Affiliations:** 1 Department of Implant Surgery, Faculty of Health Sciences, Alfonso X El Sabio University, Avda. Universidad, 1, Villanueva de la Cañada, 28691 Madrid, Spain; 2 Department of Maxillofacial Surgery, Quirón Health Hospital, 28002 Madrid, Spain; 3 Department of Surgery, Faculty of Medicine, University of Salamanca, 37008 Salamanca, Spain; 4 Department of Surgery, Faculty of Dentistry, University of Navarra, 31009 Pamplona, Navarra Spain

**Keywords:** Computer-aided surgery, Image-guided surgery, Implantology, Navigation system, Zygomatic implants

## Abstract

**Background:**

Zygomatic implants are widely used in the rehabilitation of severely atrophic maxillae, but implant placement is not without risks, and it can potentially cause damage to related anatomical structures. The aim of this study was to perform a comparative analysis of the accuracy of static navigation systems in placing zygomatic dental implants in comparison to dynamic navigation systems.

**Methods:**

Sixty zygomatic dental implants were randomly allocated to one of three study groups, categorized by which implant placement strategy was used: A: computer-aided static navigation system (n = 20) (GI); B: computer-aided dynamic navigation system (n = 20) (NI); or C: free-hand technique (n = 20) (FHI). For the computer-aided study groups, a preoperative cone-beam computed tomography (CBCT) scan of the existing situation was performed in order to plan the approach to be used during surgery. Four zygomatic dental implants were inserted in each of fifteen polyurethane stereolithographic models (n = 15), with a postoperative CBCT scan taken after the intervention. The pre- and postoperative CBCT scans were then uploaded to a software program used in dental implantology to analyze the angular deviations, apical end point, and coronal entry point. Student’s *t*-test was used to analyze the results.

**Results:**

The results found statistically significant differences in apical end-point deviations between the FHI and NI *(p* = 0.0053) and FHI and GI *(p* = 0.0004) groups. There were also statistically significant differences between the angular deviations of the FHI and GI groups *(p* = 0.0043).

**Conclusions:**

The manual free-hand technique may enable more accurate placement of zygomatic dental implants than computer-assisted surgical techniques due to the different learning curves required for each zygomatic dental implant placement techniques.

## Background

Severely atrophied, fully edentulous maxillae represent a crucial problem of esthetics and function for such patients, who often seek rehabilitation as urgently as possible. However, these procedures can be challenging for dental clinicians, as there is little available bone in which to place conventional-length implants [1]. Several proposed alternative techniques for rehabilitating atrophic maxilla include sinus lifts, grafting procedures, and augmentation techniques used to improve bone availability and better facilitate rehabilitation with implants, including for apposition grafts (with or without a Le Fort I osteotomy). These bone augmentation techniques have reported success rates ranging between 60–90% [2–4]. However, most of these techniques, including bone grafts, entail multi-stage procedures and necessitate a delayed approach, increasing the risk of postoperative complications [5]. Some clinicians have therefore suggested zygomatic dental implants as an alternative technique for rehabilitating fully edentulous maxillae without the need for any bone grafting procedures [6]. Zygomatic dental implants have been used in conjunction with conventional implants in patients presenting with severe maxilla resorption, with reported survival rates of 96–100% [7–9]. Unfortunately, potential postoperative complications can affect the maxillary sinus, particularly when zygomatic dental implants are placed inside the maxillary sinus. Sinusitis has been reported in as many as 5–6% of cases (range: 0–26.6%), although treatment with antibiotics was also shown to be widely effective in all patients [10, 11]. Research has also identified potential prosthetic complications in restorations using zygomatic dental implants, including hyperplasia, fractured fixed dental prostheses, overgrowth of the mucosa, lack of retention of overdentures, and discomfort [12]. Additionally, some studies have reported intraoperative and postoperative complications including fractured zygomatic bones, burning sensations, infection, implant fenestration, and discomfort caused by an implant protruding from under the lower eyelid [13]. It is important to improve the accuracy of zygomatic dental implant placement to reduce the risk of intraoperative and postoperative complications, particularly in severely atrophied edentulous maxillae.

In more recent years, dental implant placement is increasingly performed using image data–based navigation techniques in an effort to improve procedure outcomes and lessen the risk of potential complications [14]. This alternative surgical approach uses preoperative CBCT scans and specialized 3D implant–planning software to improve the accuracy of implant placement procedures [15]. In general, there are two different types of computer-assisted surgical implant placement techniques: dynamic navigation systems and static navigation systems. Vrielinck et al. used a surgical template modelled off a preoperative CT scan to improve the accuracy of zygomatic dental implant placement and thereby improve survival rates [16]. Chow et al. used this surgical protocol to place zygomatic dental implants with an immediate occlusal load [17]. Dynamic navigation systems can detect and monitor the placement of optical reference markers, using a tracking system array to overlay these markers over the patient and surgical instruments. Both of these navigation techniques have been extensively studied, and findings show that they greatly improve the accuracy of dental implant placement [18–20]. The mean horizontal deviation when using static navigation systems is 1.2 mm (1.04–1.44 mm) and 1.4 mm (1.28–1.58 mm) at the coronal entry point and apical end point, respectively, with a mean angular deviation of 3.5° (3.0–3.96°) [21]. Some researchers have found that deviations are lower at the at the coronal entry point (0.71 ± 0.40 mm) and apical endpoint (1.00 ± 0.49 mm), as well as there being less angular deviation (2.26 ± 1.62°), when using dynamic navigation systems [22]. However, further research is needed to corroborate these findings.

In a previous systematic review with meta-analysis conducted by the authors in 2021 [23], only one of the studies found analyzed the use of computer-assisted dynamic navigation techniques in the placement of zygomatic dental implants [24]. The number of publications about this technique has since increased [25–30], but none of them compare the results of using computer-assisted dynamic navigation techniques with those of computer-assisted static navigation techniques and freehand techniques to identify which is most accurate.

The aim of this study was to analyze and compare the accuracy of static and dynamic navigation system techniques for placing zygomatic dental implants. The null hypothesis (H_0_) states that there is no difference in the accuracy of zygomatic dental implant placement between static and dynamic navigation systems.

## Methods

### Study design

Researchers conducted a randomized controlled experimental trial based on the methodology of a previous study [31] and in accordance with International Organization for Standardization guidelines (ISO 14801). This clinical trial was conducted between January to March 2021 at the Dental Center for Innovation and Advanced Specialties at Alfonso X El Sabio University in Madrid, Spain. The study was approved by the Ethical Committee of the Faculty of Health Sciences at Alfonso X El Sabio University in December 2020 (Process no. 27/2020). The patient provided their informed consent to participate prior to using their CBCT scan in this study.

### Experimental procedure

**Fig. 1:**
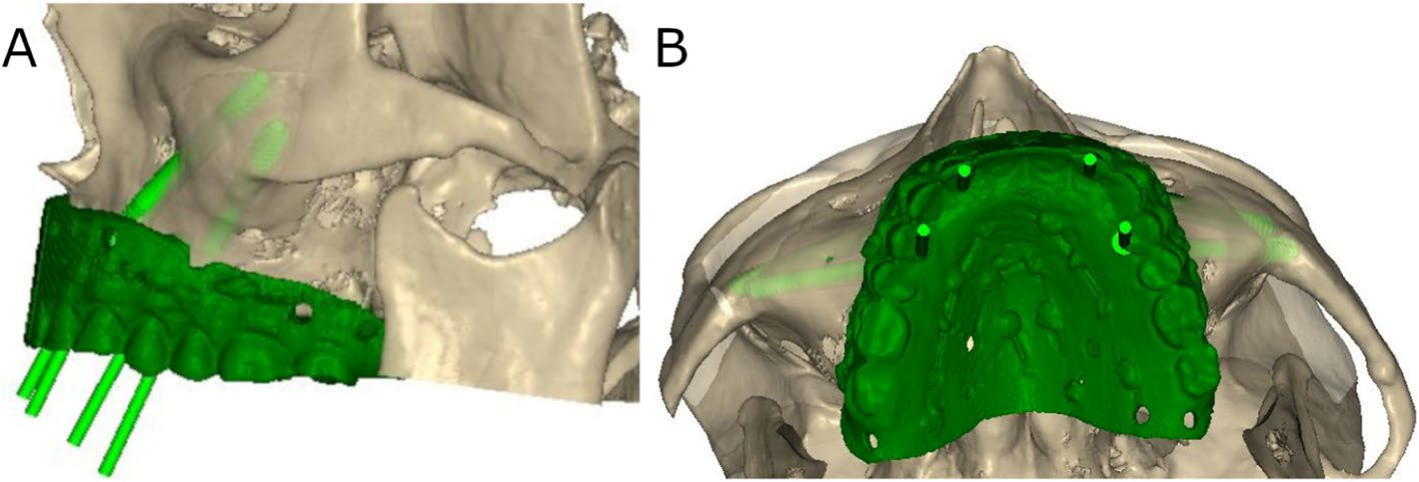
**A** Left lateral and (**B**) occlusal views of the digital mock-up used to visualize the prosthetic emergence profiles of the multiunit abutment of the zygomatic dental implants

Sixty (60) new zygomatic dental implants (IPX-Tilted System, Galimplant, Sarria, Lugo, Spain) were placed in prosthetic emergence profiles in teeth 1.2 (4.3 mm × 52,5 mm, internal taper and conical wall) (Ref.: ICMT-4352, IPX-Tilted System, Galimplant, Sarria, Lugo, Spain); 1.4 (4.3 mm × 35 mm, internal taper and conical wall) (Ref.: ICMT-4335, IPX-Tilted System, Galimplant, Sarria, Lugo, Spain); 2.2 (4.3 mm × 50 mm, internal taper and conical wall) (Ref.: ICMT-4350, IPX-Tilted System, Galimplant, Sarria, Lugo, Spain); and 2.4 (4.3 mm × 30 mm, internal taper and conical wall) (Ref.: ICMT-4330, IPX-Tilted System, Galimplant, Sarria, Lugo, Spain) following prosthetic planning (Fig. 1).

ANOVA was used to establish the sample size needed to compare the contrast null hypothesis H_0_: μ1 = μ2 = μ3, resulting in 80% power with a confidence level of 5%, with a variability between groups of 0.6 and variability within groups of 3. A preoperative CBCT scan (WhiteFox, Satelec, Merignac, France) was used to create fifteen (15) anatomically based (1:1 proportion) standardized polyurethane models of a completely edentulous, atrophic upper jaw maxilla using a stereolithographic 3D printer (Sawbones Europe AB, Malmo, Sweden). The scan was taken of a real patient using the following exposure parameters: 8.0 mA, 7.20 s, 105.0 kV peak, with a field of view of 15 mm × 13 mm.

Researchers randomized the zygomatic dental implants (Epidat 4.1, Galicia, Spain), which were allocated to one of the following study groups: A: zygomatic dental implants (Galimplant, Sarria, Lugo, Spain) placed using a computer-aided static navigation system (NemoStudio®, Nemotec, Madrid, Spain) (n = 20) (guided implant (GI)); B: zygomatic dental implants (Galimplant, Sarria, Lugo, Spain) placed using a computer-aided dynamic navigation system (Navident, ClaroNav, Toronto, Canada) (n = 20) (navigation implant (NI)); and C: zygomatic dental implants (Galimplant, Sarria, Lugo, Spain) placed manually using a freehand technique (n = 20) (freehand implant (FHI)). The zygomatic dental implants (Galimplant, Sarria, Lugo, Spain) were placed in a randomized order for all study groups (Epidat 4.1, Galicia, Spain). The order of drilling was also randomized (Epidat 4.1, Galicia, Spain), starting with the GI study group and continuing to the NI study group and FHI control group.

**Fig. 2:**
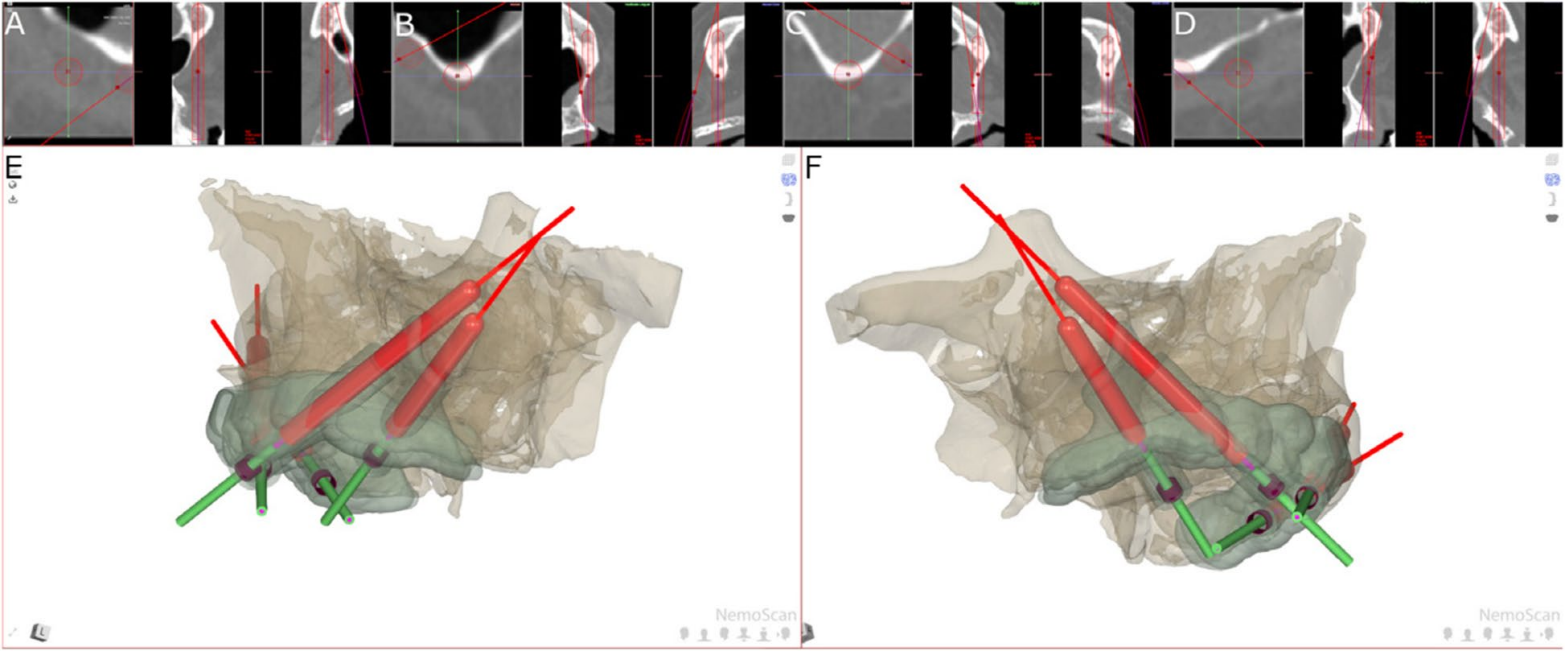
**A** Tomographic images from the zygomatic dental implants planned in teeth 2.4, **B** 2.2, **C** 1.2 and **D** 1.4 and **E** 3D reconstruction of the virtual template and the zygomatic dental implants in teeth 2.4 and 2.2 and **F** teeth 1.4 and 1.2

The zygomatic dental implants (Galimplant, Sarria, Lugo, Spain) from the GI study group were planned using 3D virtual implant-planning software (NemoScan, Nemotec, Madrid, Spain) with the measurements mentioned previously (Fig. 2A-D). After designing the virtual templates (Fig. 2E,F), they were made using a 3D printer (Objet30 OrthoDesk, Tikoa, Madrid, Spain) that polymerizes a biocompatible material (Ref.: MED610, PolyJet, Stratasys, Canada) on top of a layer of support material (Ref.: SUP 705B, PolyJet Support Material, Stratasys, Canada) using two printer heads and a lamp; one head contains the support material and the other head contains the biocompatible material. The orientation of the STL digital files was completed automatically, always leaving them as close as possible to the printing tray. For printing, the STL digital files were calculated by layers, starting with a layer of material, then filled with the support material and polymerized. Afterwards, the standardized polyurethane models were cleaned using pressurized water (WaterJet). A preliminary cleaning was carried out to eliminate all the support material, after which the models were left in a bowl of water with baking soda for one hour to completely dissolve the support material. Once the cleaning process was finished, they were cleaned again in the pressurized water machine. There was no need to place them in any curing oven since this type of printer already photo-cures the templates during manufacturing.

These templates were apt for the experimental models, and no further adjustments were necessary. Afterwards, the drilling procedure was carried out using the 2.2-mm diameter Lance Drill (Galimplant, Sarria, Lugo, Spain) at 2000 rpm under profuse irrigation to perforate the cortex and mark the direction of the zygomatic dental implants (Galimplant, Sarria, Lugo, Spain), followed by the 4-mm diameter Countersink Lateral Drill (Lindermann drill) at 2000 rpm under profuse irrigation, in accordance with manufacturer recommendations for ZAGA type 2 and type 3 IPX tilted zygomatic dental implants (Galimplant, Sarria, Lugo, Spain). Afterwards, the 2-mm diameter Drill (Galimplant, Sarria, Lugo, Spain), 3.2-mm diameter Drill (Galimplant, Sarria, Lugo, Spain), and 3.75-mm diameter Drill (Galimplant, Sarria, Lugo, Spain) were used in this order at 2000 rpm under profuse irrigation to complete the drilling protocol. Each drill in the sequence was used with its corresponding surgical template.

**Fig. 3:**
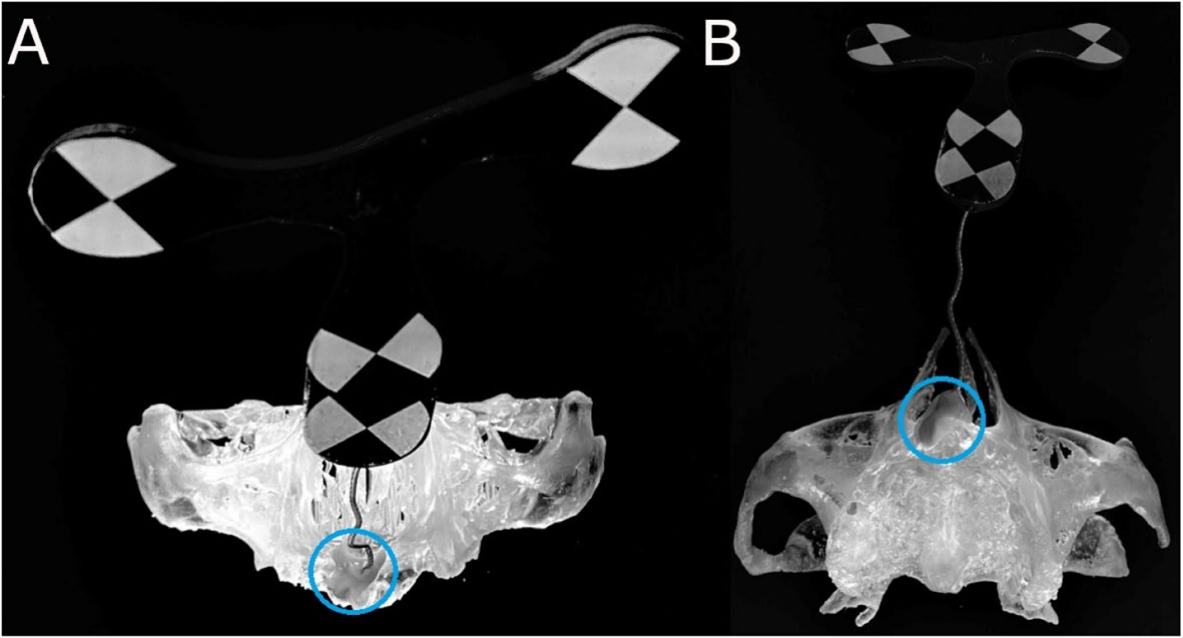
**A** Front and **B** bottom view of the black-and-white tag affixed to the surface of the anatomically based standardized polyurethane models

A preoperative CBCT scan was taken of the NI anatomically accurate standardized polyurethane models (WhiteFox, Satelec, Merignac, France) prior to placing a jaw tag. This black-and-white tag was attached to the surface of the models using a photo-polymerized composite resin (Navistent, ClaroNav, Toronto, Canada) (Fig. 3).

**Fig. 4:**
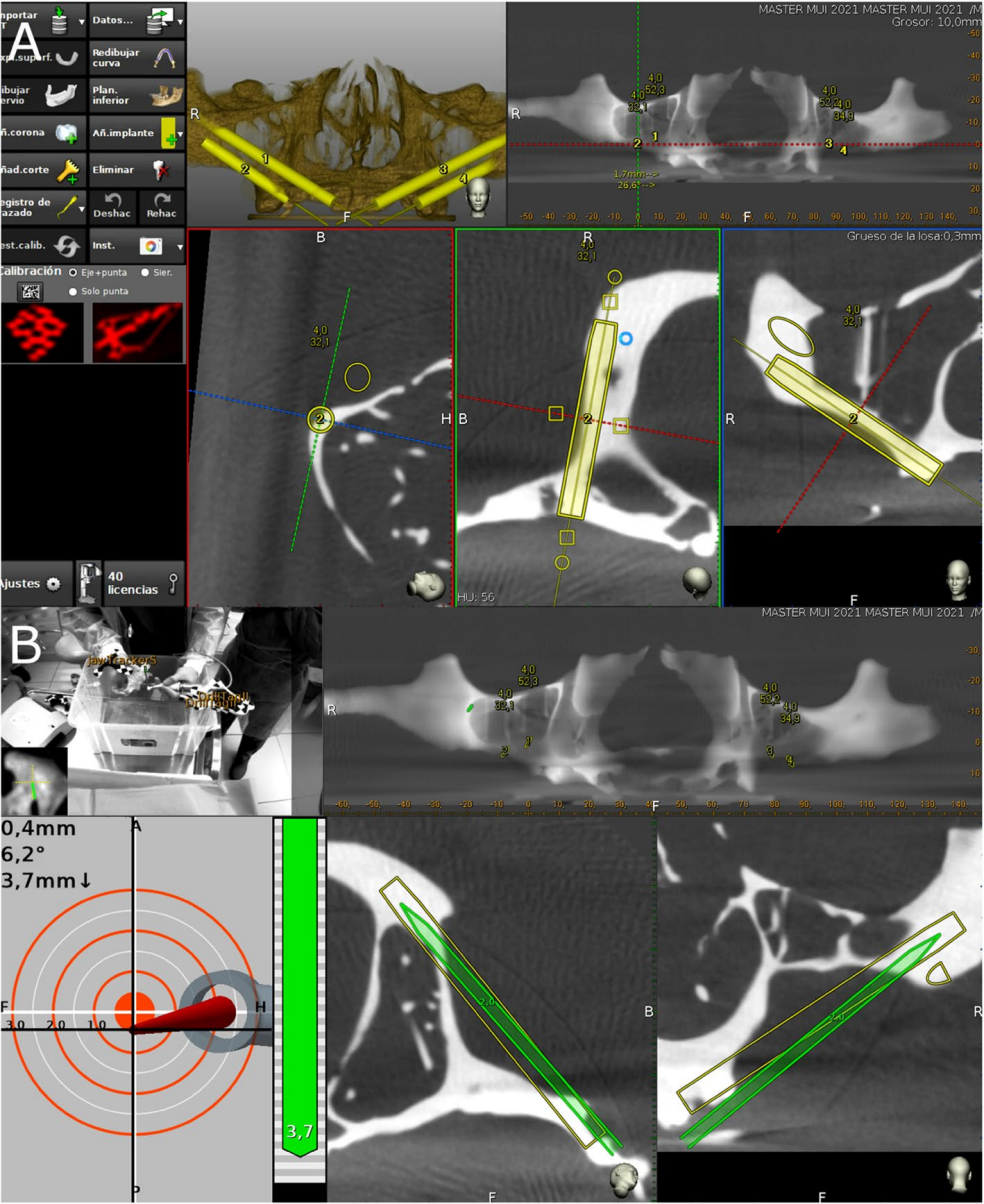
**A** Treatment-planning software preoperative planning of placement of the zygomatic dental implant for the dynamic navigation appliance and **B** capture of the real-time tracking procedure during zygomatic dental implant placement

The datasets obtained from the CBCT scan were imported into a treatment-planning software (Navident, ClaroNav, Toronto, Canada) on a laptop mounted on a mobile unit in order to recreate the implant placement previously determined during pre-surgical planning. using zygomatic anatomy guided approach (ZAGA) type 3 for the anterior zygomatic dental implants and ZAGA type 2 for the posterior zygomatic dental implants, following the classification recommended by Aparicio et al. [32, 33]. The passive optical tracking procedure and workflow of the dynamic navigation system was calibrated in the dynamic navigation system prior to the procedure by placing the drill on the black-and-white tag affixed on the surface of the anatomically accurate standardized polyurethane model (Fig. 3) to align the preoperative CBCT scan with the real environment. Afterwards, a paired-point registration based on artificial reference markers (black-and-white tags) was conducted to identify the black-and-white tag placed on the surface of the anatomically accurate standardized polyurethane models, as well as the drill tag attached to the 20:1 reduction zygoma hand piece (SZ-75, W&H, Bürmoos, Austria). The zygomatic dental implant placement was subsequently planned using the implant-planning software installed in the laptop of the dynamic navigation system. (Fig. 4A). Finally, both optical reference markers were identified and calibrated using an optical triangulation tracking system with stereoscopic motion-tracking cameras, which oriented the drilling process in real time to ensure implant placement with the planned angle, pathway, and depth. A zygomatic dental implant system (Galimplant, Sarria, Lugo, Spain) was used for the drilling, and the computer-aided dynamic navigation system was used to monitor the procedure (Fig. 4B).

**Fig. 5:**
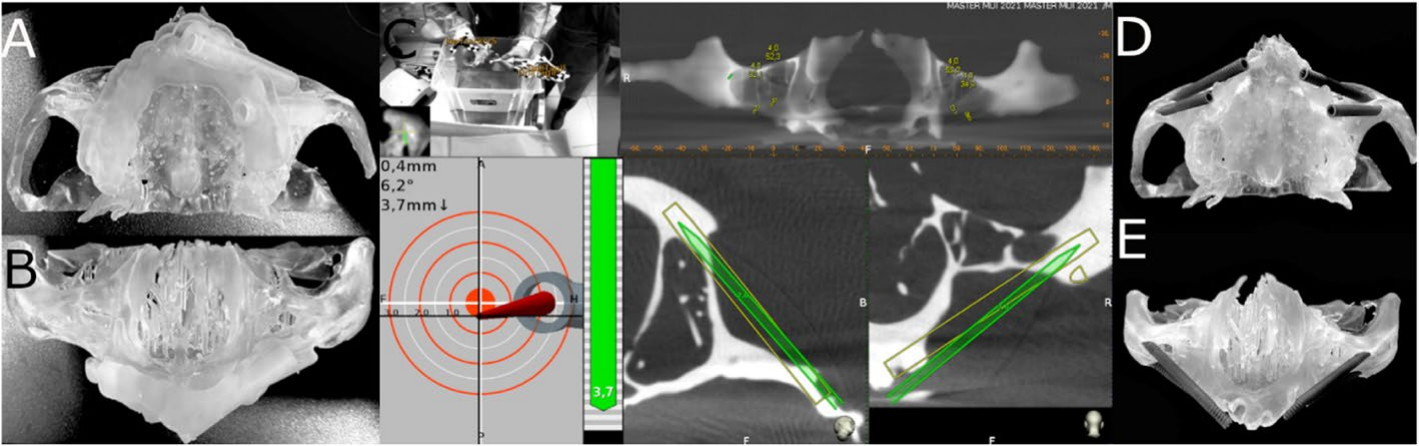
**A** Occlusal and **B** frontal view of the standardized polyurethane models with the 3D printed template for the left zygomatic dental implants. **C** Tracking procedure during zygomatic dental implant placement with the dynamic navigation appliance and **D** Occlusal and **E** frontal view of the standardized polyurethane models with the zygomatic dental implants manually placed

The zygomatic dental implants (Galimplant, Sarria, Lugo, Spain) in the FHI control group were placed manually using the CBCT scan and the preoperative planning as visual guides. An operator with previous surgical experience placed all the zygomatic dental implants (Fig. 5).

### Measurement procedure

**Fig. 6:**
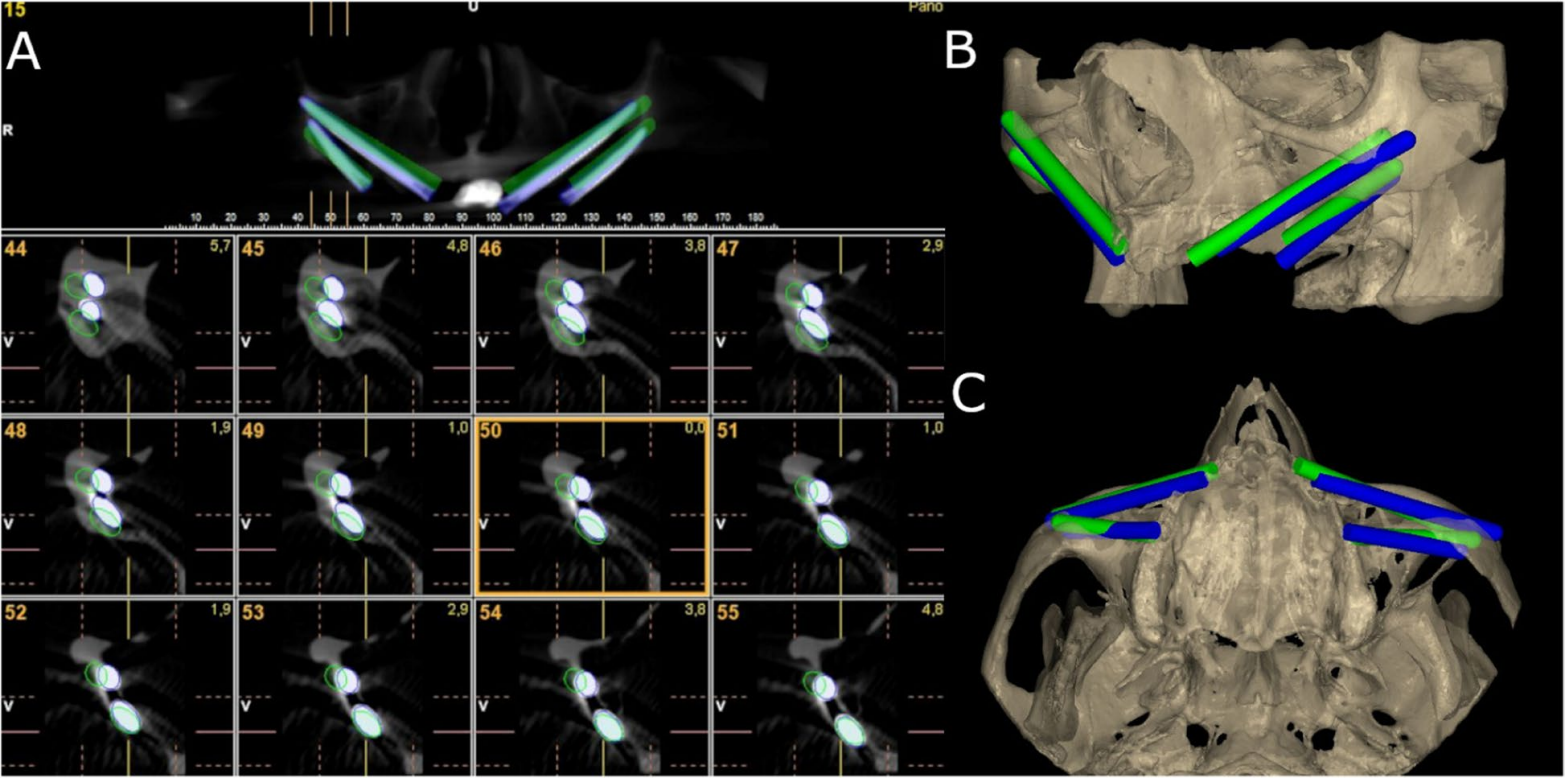
**A** CBCT images and **B**, **C** 3D render of the measurement procedure comparing the preoperatively planned (green cylinders) and postoperative implants (blue cylinders) placed on the experimental models

After placing the zygomatic dental implants, researchers took postoperative CBCT scans using the same exposure parameters described above. The CBCT scans (WhiteFox, Satelec, Merignac, France) for each study group were then uploaded into 3D implant-planning software (NemoScan, Nemotec, Madrid, Spain). Next, the post-operative CBCT scan and preoperative standard tessellation language (STL) digital file from the zygomatic dental implant planning were manually aligned by an independent operator, selecting the same anatomical key points of both the post-operative CBCT scan and the preoperative STL digital file in the 3D virtual implant-planning software (NemoScan, Nemotec, Madrid, Spain) so as to record the apical deviation, taken at the coronal entry point (mm), apical end point (mm), and angular deviation (°), the latter being measured in the center of the cylinder. This measurement procedure was used in a previous study to measure the deviations of conventional length dental implants [34]. If any deviations were noted in any of the implants, an independent operator then analyzed and compared the axial, sagittal, and coronal views (Fig. 6A–C). Researchers also noted and analyzed any deviations in the position of the zygomatic dental implants.

Afterwards, the zygomatic dental implants from both the post-operative CBCT scan and the preoperative STL digital file was virtually marked with a cylinder with the corresponding geometrical dimensions to measure the length and degrees performed using the 3D implant-planning software (NemoScan, Nemotec, Madrid, Spain) [34].

### Statistical analysis

Tables with summaries of the statistics for each of the response valuables were displayed according to group, position, and group and position: mean, median, standard deviation, number of observations, and the minimum and maximum values. These were represented visually using box plots. Linear regression models with repeated measures have been adjusted to analyze the differences by function of group, position, and the interaction between these two variables. In case of statistically significant differences, 2-to-2 comparisons were made between positions / groups. The Tukey method was used to adjust the *p*-values to correct for the type I error. SAS v9.4 (SAS Institute Inc., Cary, NC, USA) was used to carry out the statistical analysis. A significance level of 0.05 was used for statistical decisions.

## Results

Table 1 shows the mean, median, and SD values for the coronal entry point (mm), apical end point (mm) and angular deviations (°) of the GI, NI, and FHI study groups.

**Table 1:** Descriptive values of deviations at the coronal entry point (mm), apical end point (mm), and angular (°) deviations of guided implant (GI) using a static navigation system; navigation implant (NI) using a dynamic navigation system; and free-hand technique (FHI)

	n		Mean	Median	SD	Minimum	Maximum
CORONAL	GI	20	5.54^a^	5.15	1.72	2.60	8.70
	NI	19	5.43^a^	5.70	2.13	1.60	10.50
	FHI	20	4.75^a^	4.35	1.58	2.20	7.80
APICAL	GI	20	5.33^a^	5.55	2.14	1.40	9.20
	NI	19	4.92^a^	4.70	1.89	1.70	9.10
	FHI	20	3.20^b^	3.30	1.45	0.60	5.40
ANGULAR	GI	20	5.30^a^	5.35	2.80	1.30	9.70
	NI	19	7.36^a^	6.20	4.12	0.90	16.10
	FHI	20	8.47^b^	7.05	4.40	3.50	17.20

*GI* Guided implants, *NI* Navigation implants, *FHI* Free-hand implants

^a,^^b^ Statistically significant differences (*p* < 0.05) between groups

**Fig. 7:**
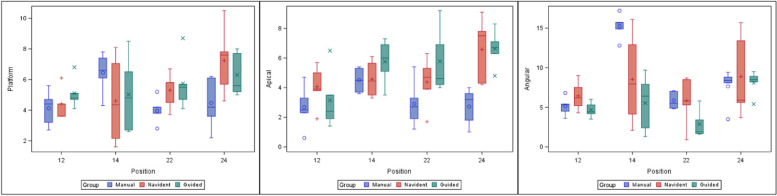
Box plot of deviations at the coronal entry point, apical end point, and angular deviations observed in the study groups and zygomatic dental implant positions. The horizontal lines in each box represent the median values

The paired Student’s *t*-test did not find any statistically significant differences, neither between study groups (*p* = 0.29065) nor in the position of the implants (*p* = 0.1312) (Fig. 7). In addition, the highest deviations were found between zygomatic dental implant positions 1.2 and 2.4 in the NI study group *(p* = 0.054).

The paired Student’s *t*-test found statistically significant differences between the FHI control group and NI study group *(p* = 0.0053) and FHI control group and GI study group *(p* = 0.0004) with regard to apical end-point deviation. No statistically significant differences were found between the NI and GI study groups *(p* = 0.6932). Statistically significant differences were found between implant positions 1.2 and 1. 4 *(p* = 0.0392) and 1.2 and 2. 4 *(p* = 0.0068). In addition, the GI study group showed higher deviations between zygomatic dental implant positions 1.2 and 2. 4 at the apical end point *(p* = 0.0068), resulting in a statistically significant difference between zygomatic dental implants placed in position 2.4 of the NI study group and FHI control group *(p* = 0.0013) and the GI study group and FHI control group *(p* = 0.0011) (Fig. 7).

The paired Student’s *t*-test found statistically significant differences between study groups with regard to angular deviation *(p* = 0.0052) and implant position *(p* < 0.001), even detecting a relationship between the study group and zygomatic dental implant position *(p* = 0.0073). Statistically significant differences were found between the FHI control group and GI study group *(p* = 0.0043); there were no statistically significant differences between the FHI control group and NI study group *(p* = 0.5335) and the GI control group and NI study group *(p* = 0.0724). There were also statistically significant differences between zygomatic dental implant positions 1.2 and 1.4 *(p* = 0.0014); 2.2 and 2.4 *(p* = 0.0178); and 2.2 and 1.4 *(p* = 0.0003), particularly in the FHI control group (Fig. 7).

To summarize, the FHI technique showed lower deviation values at the coronal entry point and apical end point. This could be due to the fact that the dental implants for the FHI control group were placed last, which meant the operator could memorize the correct position of the zygomatic dental implants. Additionally, zygomatic dental implants inserted in the posterior regions had higher deviation values at the coronal entry point, apical end point, and angular level.

One zygomatic dental implant from the NI study group was not counted, as the osteotomy site was not sufficiently stable for zygomatic dental implant placement after preparation.

## Discussion

The results of this study reject the null hypothesis (H_0_) that there is no difference in the accuracy of zygomatic dental implant placement between static and dynamic navigation systems.

These findings show that the conventional free-hand technique provides better accuracy when placing zygomatic dental implants at the coronal and apical level than the computer-aided static navigation technique and computer-aided dynamic navigation technique. However, the computer-aided static navigation technique resulted in less angular deviation than the computer-aided dynamic navigation technique and the free-hand control group. Furthermore, the zygomatic dental implants located in the anterior region showed less horizontal and angular deviation than implants in the posterior region, perhaps due to better visibility and accessibility.

Several studies have analyzed the use of computer-aided static navigation techniques in zygomatic dental implant placement. Findings by Vrielinck et al. showed an average coronal entry point of 2.77 ± 1.61 mm, apical end point of 4.46 ± 3.16 mm, and angular deviation of 5.14 ± 2.59° [16]. The accuracy of computer-aided static navigation when used in conjunction with conventional-length dental implants has also been widely studied. Hoffmann et al. found statistically significant differences in accuracy between implants placed using computer-aided dynamic navigation systems and those placed using the conventional free-hand technique, with mean angular deviations of 4.2 ± 1.8° and 11.2 ± 5°, respectively [35]. Chen et al. found similar horizontal deviation values at the apical end point when using a computer-aided dynamic navigation system (1.35 ± 0.55 mm), a computer-aided static navigation system (1.50 ± 0.79 mm), and free-hand implant placement (2 ± 0.79 mm). Higher angular deviation values were observed when using the computer-aided dynamic navigation system (4.45 ± 1.97°), computer-aided static navigation system (6.02 ± 3.71°), and free-hand implant placement (9.26 ± 3.62°) [36]. Wu et al. reported an average coronal entry point of 1.57 ± 0.71 mm, apical end point of 2.1 ± 0.94 mm, and angular deviation of 2.68° ± 1.25° in two hundred and thirty-one zygomatic dental implants placed using a dynamic navigation system [23]. Tao et al. reported that the dynamic navigation technique is influenced by radio-diagnostic technique; specifically at the apical deviation level (*p* < 0.001), with the CBCT resulting in higher values for accuracy than the conventional multi-slice CT [37]. In summary, the dynamic navigation systems have become very helpful in transferring the surgical plan to the patient and avoiding complications of zygomatic dental implant placement for the reconstruction of severe maxillary atrophy and maxillary deficiency defects [38].

The present study did not find any statistically significant differences between computer-aided static and dynamic navigation techniques at the coronal *(p* = 0.2904), apical *(p* = 0.8309), and angular level *(p* = 0.1410); this may be due to the learning curve for using computer-aided dynamic navigation systems, which could affect results. In other words, the operator acquired experience as they placed the 40 zygomatic dental implants in the anatomically identical experimental models, resulting in the operator having more accurate knowledge in the last study group (FHM). Spille et al. reported statistically significant differences at the apical level (*p* < 0.001) and angular level (*p* < 0.001) of implants placed using a dynamic navigation system after two weeks of learning [39]. Wang et al. analyzed the learning curve of two dynamic navigation systems, which approached each other after 12 dental implants, and finally converged after 27 dental implants [40]. Marques-Guasch et al. observed a trend towards improvement in accuracy between implants 8 and 17 placed using a dynamic navigation system [41], and Sun et al. showed a stabilization of the learning curve after placing five dental implants using a dynamic navigation system [42]. However, Cassetta et al. did not identify a “learning curve” effect for dental implants placed using a static navigation system [43]. Finally, the potential difference in surgical accuracy of the three drilling techniques, dynamic navigation systems, static navigation systems, and freehand drilling, was highlighted in a study on cast models by Chen et al. [36], who reported that the dynamic navigation system exhibited higher accuracy at the apical and angular level. A high level of attention and more time are required from both the clinician and technician for appropriate setup; this is associated with a learning curve [22, 44]. The point-based registration process used in the dynamic navigation systems by identifying fiducial markers (black-and-white tags) is prone to mistakes, such as fiducial localization error (FLE), fiducial registration error (FRE), and target registration error (TRE) [45, 46]. FLE refers to an incorrect identification of fiducial markers by the computer software of the dynamic navigation system, which is dependent on the image voxel size and size of the fiducial markers. FRE is related to the root mean square between adjacent fiducial markers. Finally, TRE refers to the discrepancy between the coordinator of the navigated surgical tool and the corresponding coordinator of the surgical target, which is of vital importance for safely and precisely performing the surgery [47]. Therefore, implanted bone-anchored screws have been shown to be the most accurate fiducial markers and are regarded as the gold standard for point-to-point registration [48].

The results of the present study corroborate those of the study by Mediavilla-Guzman et al., who compared the accuracy of computer-aided static and dynamic navigation systems in the placement of conventional-length dental implants; they found no statistically significant differences between computer-aided static and dynamic navigation systems at the coronal (*p* = 0.6535) and apical (*p* = 0.9081) levels. They did find statistically significant differences between the angular deviations of computer-aided static and dynamic navigation systems *(p* = 0.0272) [20]. The present study found higher horizontal deviations owing to the longer implant length of zygomatic dental implants, particularly at the apical end point. The accuracy of computer-aided static navigation techniques for dental implant placement directly depends on the design and manufacturing process used to create the surgical template, and inaccurate manufacturing can potentially lead to intraoperative complications [14]. Computer-aided dynamic navigation systems provide clinicians with a direct view of the surgical field and enable them to adjust the position of an implant if needed [16, 22]. Additionally, these systems are especially useful in patients with mouth openings or in cases of rehabilitation of the posterior region [18, 21]. The primary drawback of computer-aided dynamic navigation systems is that it is difficult to keep sight of the dynamic navigation system display throughout the entire procedure. As a result, augmented reality devices are often used to project a virtual image of the computer-aided dynamic navigation system, enabling the clinician to maintain visibility of the surgical field [49, 50]. The accuracy of the depth, angle, and position of implant placement can be compared across image-guided navigation systems. This is necessary to reduce the risk of intraoperative surgical complications and enable clinicians to properly position dental implants, which is crucial because poor positioning can result in compromised primary stability and negatively affect restoration techniques with immediate loading [16, 18, 21]. These techniques also prevent the need for the large excisions sometimes performed to reveal the implant platform after the healing process, instead providing a transgingival, minimally invasive approach for implant placement [18, 36]. These techniques may prove particularly useful in high-risk patients, including cardiovascular patients who take anticoagulation medications and patients with atrophic, edentulous mandibles [18].

This in vitro study is somewhat limited in scope due to its experimental nature. That being said, the methodology used can be easily applied to clinical studies, and it can be used to provide evidence with a view to determining the most accurate technique for zygomatic dental implant placement.

## Conclusion

Bearing in mind the limitations of this study, the results of the present study indicate that the manual free-hand technique results in more accurate placement of zygomatic dental implants than computer-assisted surgical techniques due to the relevance of the learning curve effect inherent to each zygomatic dental implant placement technique. Additionally, the results of the present study found that placement of zygomatic dental implants is more accurate in the anterior region than in the posterior region; further studies are needed to these results.
